# Mutational Landscape of the BAP1 Locus Reveals an Intrinsic Control to Regulate the miRNA Network and the Binding of Protein Complexes in Uveal Melanoma

**DOI:** 10.3390/cancers11101600

**Published:** 2019-10-19

**Authors:** Amit Sharma, Arijit Biswas, Hongde Liu, Sagnik Sen, Anoosha Paruchuri, Panagiotis Katsonis, Olivier Lichtarge, Tikam Chand Dakal, Ujjwal Maulik, M. Michael Gromiha, Sanghamitra Bandyopadhyay, Michael Ludwig, Frank G. Holz, Karin U. Loeffler, Martina C. Herwig-Carl

**Affiliations:** 1 Department of Ophthalmology, University Hospital Bonn, 53127 Bonn, Germany; Amit.Sharma@ukbonn.de (A.S.); Frank.Holz@ukbonn.de (F.G.H.); Karinloeffler@uni-bonn.de (K.U.L.); 2 Institute of Experimental Hematology and Transfusion Medicine, University Clinic of Bonn, 53127 Bonn, Germany; arijit.biswas@ukbonn.de; 3 State Key Laboratory of Bioelectronics, Southeast University, Nanjing 210096, China; liuhongde@seu.edu.cn; 4 Department of Computer Science and Engineering, Jadavpur University, Kolkata 700032, India; sagnik.sen2008@gmail.com (S.S.); umaulik@cse.jdvu.ac.in (U.M.); 5 Department of Internal Medicine, Division of Medical Oncology and Comprehensive Cancer Center, The Ohio State University, Columbus, OH 43210, USA; anooshaparuchurii@gmail.com; 6 Computational and Integrative Biomedical Research Center, Baylor College of Medicine, Houston, TX 77030, USA; katsonis@bcm.edu (P.K.); lichtarg@bcm.edu (O.L.); 7 Department of Biotechnology, Mohanlal Sukhadia University, Rajasthan 313001, India; tikam260707@gmail.com; 8 Department of Biotechnology, Bhupat and Jyoti Mehta School of BioSciences, Indian Institute of Technology Madras, Chennai, Tamilnadu 600036, India; gromiha@iitm.ac.in; 9 Indian Statistical Institute, Kolkata, West Bengal 700108, India; sanghami@gmail.com; 10 Department of Clinical Chemistry and Clinical Pharmacology, University Hospital of Bonn, 53127 Bonn, Germany; mludwig@uni-bonn.de

**Keywords:** BAP1, uveal melanoma, UCH domain, miRNAs, TCGA, HCF-1, FOXK2, ASXL1, BARD1, BRCA1, evolutionary action score

## Abstract

The *BAP1* (BRCA1-associated protein 1) gene is associated with a variety of human cancers. With its gene product being a nuclear ubiquitin carboxy-terminal hydrolase with deubiquitinase activity, *BAP1* acts as a tumor suppressor gene with potential pleiotropic effects in multiple tumor types. Herein, we focused specifically on uveal melanoma (UM) in which *BAP1* mutations are associated with a metastasizing phenotype and decreased survival rates. We identified the ubiquitin carboxyl hydrolase (UCH) domain as a major hotspot region for the pathogenic mutations with a high evolutionary action (EA) score. This also includes the mutations at conserved catalytic sites and the ones overlapping with the phosphorylation residues. Computational protein interaction studies revealed that distant BAP1-associated protein complexes (FOXK2, ASXL1, BARD1, BRCA1) could be directly impacted by this mutation paradigm. We also described the conformational transition related to BAP1-BRCA-BARD1 complex, which may pose critical implications for mutations, especially at the docking interfaces of these three proteins. The mutations affect - independent of being somatic or germline - the binding affinity of miRNAs embedded within the *BAP1* locus, thereby altering the unique regulatory network. Apart from UM, BAP1 gene expression and survival associations were found to be predictive for the prognosis in several (*n* = 29) other cancer types. Herein, we suggest that although BAP1 is conceptually a driver gene in UM, it might contribute through its interaction partners and its regulatory miRNA network to various aspects of cancer. Taken together, these findings will pave the way to evaluate BAP1 in a variety of other human cancers with a shared mutational spectrum.

## 1. Introduction 

*BAP1* (BRCA1-associated protein 1) with its gene product acting as a deubiquitinating enzyme, is a critical tumor suppressor gene that is mutated in various human cancers, including metastatic uveal melanoma, pleural mesothelioma, and renal cell carcinoma [1,2,3,4]. Germline *BAP1* mutations define the recently identified BAP1 cancer syndrome with affected patients developing different tumor entities, such as uveal and cutaneous melanoma, malignant mesothelioma, atypical Spitz tumors and others. The apparent ability of BAP1 mutations, both somatic and germline variants, to cause multiple tumor types suggests that this gene has a major role in influencing cancer cell growth [5].

BAP1 interacts with several chromatin-modifying factors and transcriptional regulators. There is, however, a paucity of knowledge about the exact molecular dynamics for its function. Particularly, in uveal melanoma (UM), the most frequent intraocular tumor of the eye, *BAP1* mutations were identified in 84% of tumors with a metastasizing phenotype [2]. While a wild type *BAP1* is associated with disomy of chromosome 3 (D3) and a low risk for metastasis in UM *BAP1* mutations follow the appearance of monsomy of chromosome 3 (M3) and with a high probability for metastasis [6,7]. Immunohistochemically, BAP1 mutations are characterized by absence of nuclear staining (but commonly cytoplasmic staining), while BAP1 wildtype shows a strong nuclear staining reaction in the tumor cells. Recently, a novel BAP1 cytoplasmic function was introduced, showing that ER-localized BAP1 can bind and stabilize IP3R3 to modulate Ca2+ release resulting in apoptosis [8].

Currently, the D3/M3 status or gene expression panels can be used to determine the risk for metastasis in UM patients. Other genetic alterations, such as deletion/duplication of driver genes (GNAQ, GNA11, EIFAX, SF3B1, and BPA1) also act as additional contributors towards the UM specific clinical evaluations [9,10]. Aberrant miRNA expression was found in UM and allowed for a differentiation between high risk and low-/intermediate-risk tumors in a recent study [11]. However, due to population-specific genetic effects, the approach of using miRNAs for the risk stratification of UM is still a challenge. In a few other studies using the TCGA UM (UVM), database miRNA dysregulation was observed. However, a concordance is only seen for a few miRNAs [12,13,14]. Although somatic mutations of the *BAP1* gene are quite prevalent in UM, the impact of these mutations on the structural architecture of the BAP1 protein and its associated complexes has not been reported, yet.

Herein, we have created a comprehensive map of all reported UM-associated somatic and germline missense mutations located in the *BAP1* gene and have analyzed their structural and evolutionary impact. In addition, we investigated the potential consequences of these mutations on the miRNAs embedded in the 3ÚTR region of the *BAP1* gene. Furthermore, we determined the distinctive gene expression spectra of the BAP1 gene in different types of cancer.

## 2. Material and Methods 

### 2.1. BAP1 Mutational Landscapes and Structural (Insilco) Analysis 

Human *BAP1* (chromosome 3p21.1) gene sequence was taken from UCSC genome browser (hg/19). Subsequently, 34 missense mutations selected from a literature search were mapped accordingly. The BAP1 protein has not been characterized biophysically, and there is no complete structure for the protein in the PDB database. Therefore, in order to gain structural functional insights into the mutational landscape of BAP1, we used modeling and docking strategies. The BAP1 protein was modelled on the ITASSER server (https://zhanglab.ccmb.med.umich.edu/I-TASSER/) using the server´s default conditions [15]. The model with the best C-score (−0.52) was chosen as the model of choice for further investigations. This model was then docked to structures of five known interacting partners of BAP1, i.e., Asxl1, FoxK2, BRCA1, BARD1 and HCF-1. Amongst the interacting partners, we used biophysical structures downloaded from the PDB database (if available like in the case of the BRCA1-BARD1 heterodimeric complex: PDB ID: 1JM7; NMR structure) [16]. In the absence of biophysical structures, models of the protein, or its putative interacting region/domain were downloaded from Modbase database (https://modbase.compbio.ucsf.edu/modbase-cgi/index.cgi) [17]. The docking was performed on the Z-dock rigid docking server (http://zdock.umassmed.edu/) [18]. During docking, non-interacting regions of either bimolecular interaction partners (known from previous biochemical/biophysical evidence) were excluded. The topmost 10 docks from each dock run were critically analyzed, and the model with the best docking score was evaluated for the involvement of the residues on which mutations have been reported within the inter-subunit interfaces. Furthermore, to analyze the structure flexibility of our BAP1 model, it was subjected to coarse-grained protein structure dynamics and normal mode analysis on the CABS 2.0 and iMODS server, respectively [19,20]. The PDB co-ordinates for the BAP1 model were submitted to both servers under default conditions. The output files from both servers were analyzed to predict the collective functional motion of the protein, especially with respect to its individual domains. 

Phosphorylation sites within the BAP1 sequence with a prediction score ≥ 0.5 were determined from NetPhos 3.1 Server (http://www.cbs.dtu.dk/services/NetPhos/) [21,22]. In addition, 17 kinases were also evaluated that could possibly phosphorylate sites in the BAP1 sequence (Table S2).

### 2.2. Evolutionary Action (EA) Score

To determine the pathogenic probability of *BAP1* mutations, we used the Evolutionary Action (EA) scores [23]. EA relies on a formal equation of the genotype-phenotype relationship that estimates the fitness effect of each mutation [24,25]. Briefly, the percentile rank of each variant is calculated in the scale of 0 (benign) to 100 (pathogenic). Usually, the loss of function mutations typically have high EA scores (e.g., >70), benign mutations have low EA scores (e.g., <30), while intermediate EA scores (~30–70) may not deactivate the protein, but rather have either detrimental or gain of function effect to the overall function.

### 2.3. MiRNA Analysis and Transcription Factor Regulatory Networks

To define the binding affinity of the miRNAs embedded in its 3′ UTR towards BAP1 mutations, we implemented an RNA sequence-based statistical approach (RNA hybrid) [26,27]. Briefly, the energetically most favorable binding conformations were predicted, and minimum free energy (mfe) of the BAP1 mutants—miRNAs were calculated (units:kcal/mol). The values with statistical significance (*p* ≤ 0.05) were considered for analysis. BAP1-miRNA sequences were derived from UCSC target scan (www.targetscan.org), and UM-associated miRNA were searched by literature mining. We obtained TF-miRNA-Gene regulatory network information using TransmiR V2.0 [28] and cytoscape 3.7.0 [29] with the highest correlation (*p* ≤ 0.05). Gene ontology (GO) analyses were conducted by DIANA-miRPath V3.0 [30] by identifying a stringent list of annotation corresponding to the selected miRNA. 

### 2.4. BAP1 Gene Expression Datasets and Prognostic/Diagnostic Estimations

The Cancer Genome Atlas (TCGA) project was used to retrieve information regarding BAP1 expression, patient survival and mutational data, mainly characterized from the initial release of Genomic Data Commons (GDC) in October 2016 (https://portal.gdc.cancer.gov/) using RTCGAToolbox [31]. A total of 9523 samples across 29 tumor types were downloaded, including 8811 tumor tissues and 712 non-tumor tissues. In the data, gene expression is represented with fragments per kilobase million (FPKM). Genes whose expression is associated with a differential overall survival were identified with a log-rank test in a Kaplan–Meier survival model. In each cancer type, patients were classified into two groups (high(H)- and low(L)-expression group) using the expression median of the gene as a cutoff. The survival difference was also tested for these two (H/L) groups. The area under the receiver operating characteristic (ROC) curve (AUC) was employed to indicate the diagnostic capacity of the gene for control and cancer samples. The detail nomenclatures for 29 tumor types used to analyze BAP1 expression are shown in the abbreviation list. 

## 3. Results

### 3.1. Interpretation of BAP1 Variants and the Respective Evolutionary Action Score 

**Figure 1:**
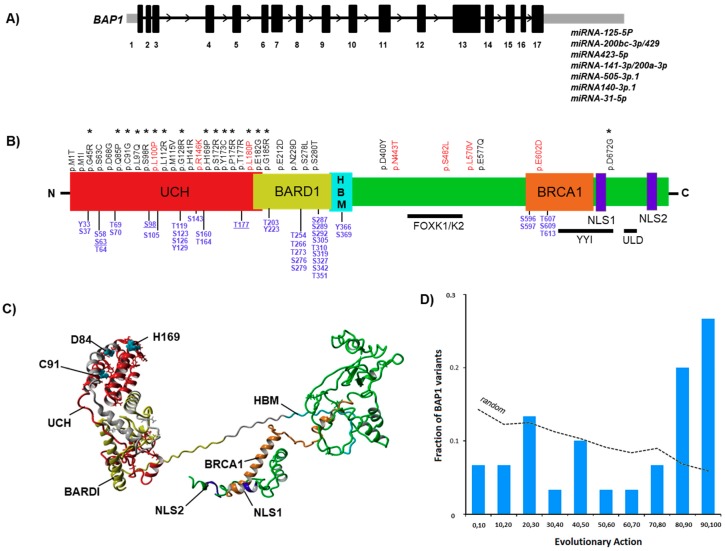
BAP1 mutational landscape and determinants of the protein structure. (**A**) Gene structure and miRNAs embedded in the 5´UTR region of *BAP1*. (**B**) Schematic structure of BAP1 domains with precise locations of UM-associated missense mutations (Somatic: Black; Germline: Red) and the phosphorylation residues (blue). Mutations with high evolutionary action (EA) score are marked by an asterisk. (**C**) The BAP1 structural model is shown in ribbon format with individual domains and regions of interest colored differently and labelled. The reported mutated residues are depicted as stick models, while the active site residues are depicted in ball models. (**D**) Plot showing EA score of the BAP1 variants (bars) and the fraction of the 4772 random missense BAP1 variants (dotted line).

Herein, we focused on 34 BAP1 specific missense mutations which were previously shown to be associated with UM (Figure 1B). The majority of them (27/34) were somatic, while few (7/34) of them were germline mutations. The ubiquitin carboxyl hydrolase (UCH) domain (including the BARD1 interaction region) of the BAP1 protein emerged as a hotspot region for missense mutations. Independent of a somatic or germline mutation type, most of them (15/27) showed high evolutionary action (EA) scores (Figure 1B). Importantly, among the conserved UCH residues (C91, H169, and D184), two mutations (C91G, H169P) showed a high EA score. A comparison to 4,772 missense BAP1 variants resulting from all possible nucleotide changes, the observed BAP1 variants were significantly skewed (Kolmogorov-Smirnov *p*-value of 10^−4^) to EA scores of 80–100 predicting them to be highly pathogenic (Figure1D, Table S1). Full scan of the BAP1 protein also revealed 17 phosphorylation sites in the UCH domain. Three mutations (S63C, S98R, T117R) were found to be overlapping with these phosphorylation sites (Figure 1B, Figure S2, Table S1). 

### 3.2. In Silico Characterization and Structural Modeling of the BAP1 Protein

BAP1 model obtained from the ITASSER (C-score: −0.52) showed a clear structural division into ordered N and C terminal regions linked by a disordered 74 amino acid long stretch (residues 336–410) (Figure 1C). The C-terminal region appears to be folded back towards the N-terminal region together with parts of the BRCA1 interacting domain, hence, making non-covalent contacts with the disordered region. This disordered stretch appears to be very critical for the conformational availability of BAP1 protein-protein interaction surfaces, owing, therefore, to its flexibility. The disordered region also comprises the HCF1 binding motif (HBM) domain residues 366–369 which are required for the interaction with HCF-1. In the ordered region, both N and C terminal are dominated by helixes with only a single three anti-parallel beta sheeted arrangement observed at the C-terminal region. The beta sheeted region lies in a part of the protein that has not as yet been functionally assigned to a particular domain. The overlapping UCH and BARD1 domains are dominated by one major four-helix bundle (exclusively in the UCH domain) and two overlapping three-helix bundles. The three major active site residues critical to the enzymatic activity of BAP1 lie on the four-helix bundle with their side chains oriented towards the sterically close-packed hydrophobic core of this helix bundle. In addition, the C-terminal BRCA1 interacting domain is split into an ordered helix and a semi-helical region. The ordered helix is part of a three-helix bundle that extends beyond the BRCA1 interacting domain and carries two nuclear localization signals of BAP1. 

### 3.3. Effect of Mutations on the Main Fold of BAP1 

A high percentage of the BAP1 mutations were observed in the UCH domain. A number of these mutations comprise substitutions to amino acids like glycine and proline (D68G, Q85P, C91G, L100P, H169P, L180, PE182G) that will result in disruption of the helices and exposure of the hydrophobic core to solvents, thereby leading to the unfolding of this domain. The mutation C91 in the UCH region is substituted to cysteines which could result in the formation of native disulfide bonds and alters the conformational landscape of the UCH domain, therefore, deleteriously affecting the enzymatic efficiency. C91 has been previously shown to ablate enzymatic activity without interfering with its interactions with BRCA1 or with FOXK1/FOXK2. The three germline mutations (L100P, R146K, L180P) occurring on the UCH domain are semi-conserved in their biophysical and biochemical nature, and therefore, are expected to have less deleterious consequences on this domain. A number of mutations also involve the substitution of a hydrophobic residue to a large polar charged residue like arginine (G45R, S98R, L112R, G128R, H141R, S172R, P175R, T177R, G185R) which will disturb the closely packed hydrophobic core of the UCH domain and contribute to the unfolding of the domain structure. As compared to the mutations in the N-terminus, the mutations at the C-terminus (D672G, E602D) are few and occur mostly on the structurally disordered regions, and their impact most likely would be on either structural flexibility or on the interaction of BAP1 with other proteins. We had earlier reported one C-terminal mutation (c.2001delG; p.[Thr668Profs*24] close to the nuclear localization signals (NLS) region and have shown the structural changes in the BAP1 protein [32].

### 3.4. Effect of Mutations on the Interaction of BAP1 with Other Proteins

**Figure 2:**
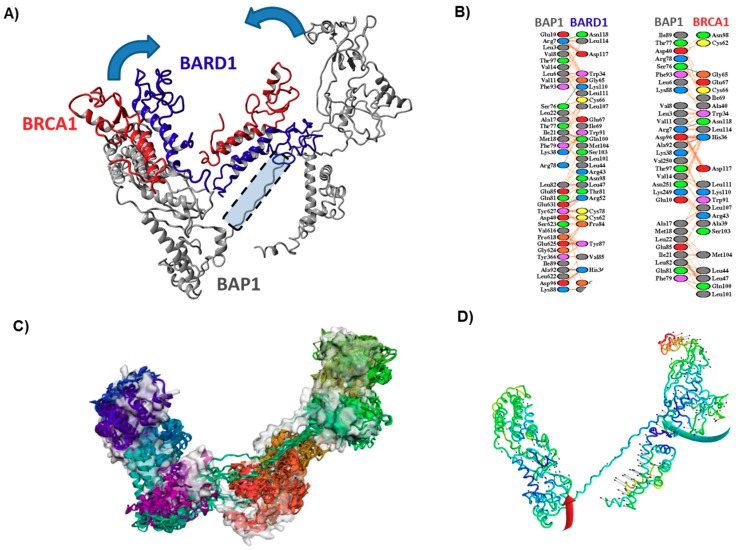
Conformational transition related to BAP1-BRCA-BARD1 complex. (**A**) The BAP1 structural model depicted with two select high scoring BRCA-BARD1 complex docking poses each docked with restraints specific to BRCA1 (left) and BARD1 (right). The structure is illustrated in ribbon format with BAP1 colored grey, while BRCA1 and BARD1 colored red and blue, respectively. The intervening disordered region that serves as a hinge for the conformational transition has been shaded and marked. The proposed movement of the N and C terminal regions of BAP1 has been shown with arrows (**B**) The inter-residue contacts between BAP1-BARD1 and BAP1-BRCA1 as observed in the two high scoring docking poses shown in (**A**,**C**,**D**). The putative motion as observed from the output files of the CABS-Flex and iMODS server, respectively. Both BAP1 structures are depicted in ribbon format and colored domain-wise based on inter-region movement. In (**C**), the starting structure is also depicted by its surface area (grey colored). In (**D**), large arrows depict the movement of the domains in the perspective of the global fold of the protein, while small arrows depict subtle inter-domain movements.

To define the consequences of these mutations, we also generated protein models from BAP1 associated proteins and performed docking studies. To begin with, the poses generated by docking the BRCA1-BARD1 heterodimeric crystal structure on the BAP1 model resulted in an interesting pattern which further implicates the disordered amino stretch between the ordered regions of BAP1 as a critical region for protein flexibility (Figure 2, Figure S1). The docking poses when were generated by including restraints specific to BRCA1 generated poses clustered around the BRCA1 interacting domain of BAP1. Similarly, docking poses resulting from restraints specific to BARD1 generated poses clustered around the BARD1 domain of BAP1. Since it is the ring finger domain of both BARD and BRCA1 that interacts with BAP1, it is essential that the BARD1 and BRCA1 interacting region be spatially close. However, in our BAP1 model, these two regions are not in close proximity. They are separated by the disordered region that we have elaborated on earlier. Structural flexibility analysis performed on the BAP1 model suggests that the protein would twist at both C and N terminal ends with the disordered stretch acting as a structural hinge for doing so (Figure 2A). Therefore, the BAP1-BRCA-BARD1 complex results in a conformational transition, pulling the relatively rigid N and C terminal regions closer to each other. This conformational transition could have critical implications for mutations especially at the docking interfaces of all three proteins, or even at the domain boundaries of BAP1 that could potentially hinder the complex formation (E182G, G185R, E212D, N229D, S278L, S280T, E602D). At the N-terminus, two mutations (E182G, G185R) showed specificity towards the BARD1 interaction site (Figure 2B). The C-terminus harbors very few mutations; however, three among them were located on the positions used by BAP1 to interact with other distant proteins, such as D672 (YY1), E602D (BRCA1), and S482L (FOXK2). A very limited number of mutations (S482L, D672G, E182G, G185R, E602D) were observed in the putative interface regions of BAP1 (Figure 2C,D, Figure S1). These mutations also either act as a helix breaker or result in the changes in electrostatic polarity (except E602D). However, all of them harbor the potential to alter the putative protein-protein interaction interfaces of BAP1. Only one of the C-terminal BAP1 mutations lies in the BRCA1 interaction domain and might have implications in its putative interaction with BRCA1, even though the wild type and the substituted amino acid, in this case, are similar in nature. 

### 3.5. Impact of Variants on the Binding Affinity of BAP1-Associated miRNAs

**Figure 3:**
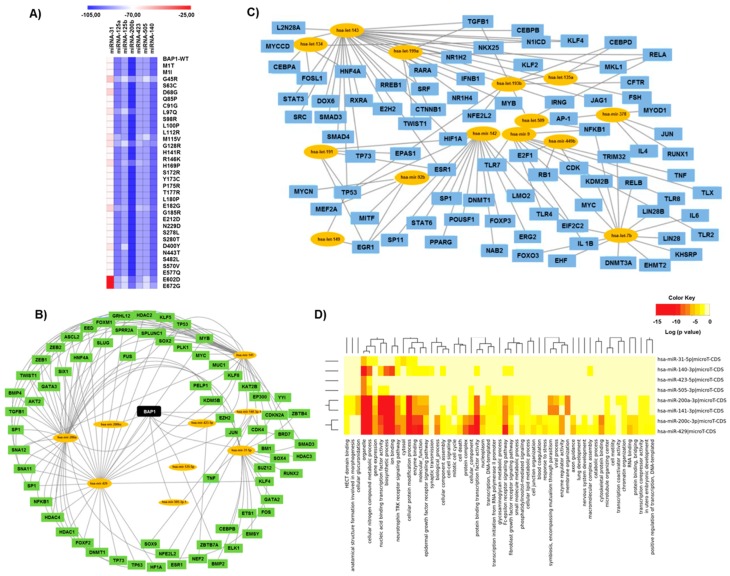
BAP1-miRNAs and associated gene regulatory network. (**A**) Heatmap showing fold change values of minimum free *binding* energy (*mfe*) intensity for BAP1 variants and the miRNAs (units: kcal/mol). (**B**) Local gene regulatory network associated with BAP1 and miRNAs. (**C**) Global gene regulatory network associated with miRNAs known to be altered in UM. (**D**) Gene ontology terms associated with BAP1-miRNAs.

A miRNA cluster was found to be embedded in the 3′UTR region of the *BAP1* gene (Figure 1A). To determine the impact of BAP1 mutants on these miRNAs, we calculated the respective binding affinities for each variant versus individual miRNAs. All UM associated mutations showed variable binding affinities for these miRNAs. However, D672G and E602D were found to have a higher minimum free binding energy (mfe) as compared to other mutations (Figure 3A, Table S2). The binding affinity of C-terminal mutations was stronger than for mutations at the N-terminus which was evident from the seven mutations (G45R, Q85P, S98R, G128R, H141R, S172R, G185R) located in the UCH domain showing a varying range of the mfe.

Further analysis revealed 69 target genes associated with these BAP1-associated miRNAs (Figure 3B), which were different from the targets globally effected by UM associated miRNAs (Figure 3C). We identified several chromatin-associated genes as putative targets, and among them, histone deacetylates (HDAC1, HDAC2, HDAC3, HDAC4) appear to be highly regulated through BAP1-miRNAs. In addition, DNA methyltransferases/DNMTS (DNMT1, DNMT3) also show a minor association with this network. All predicted target genes were checked for enrichment analysis and several cancer-related pathways were signified (adjusted *p* ≤ 0.01) (Figure 3D). Overall, the analysis showed that the regulatory network associated with BAP1-miRNAs is distinct from the global miRNAs regulatory network associated with UM. Hence, one may speculate about the local impact of these mutations on the BAP1-associated miRNA network. We also investigated and found no long-noncoding RNA (lncRNAs) overlapping the BAP1 gene.

### 3.6. BAP1 Gene Expression Penetrance and Prevalence in Cancer Spectrum

**Figure 4:**
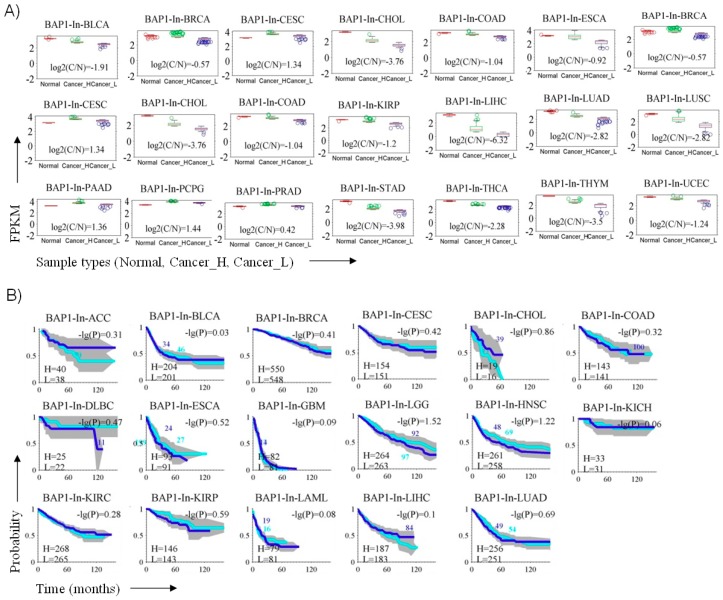

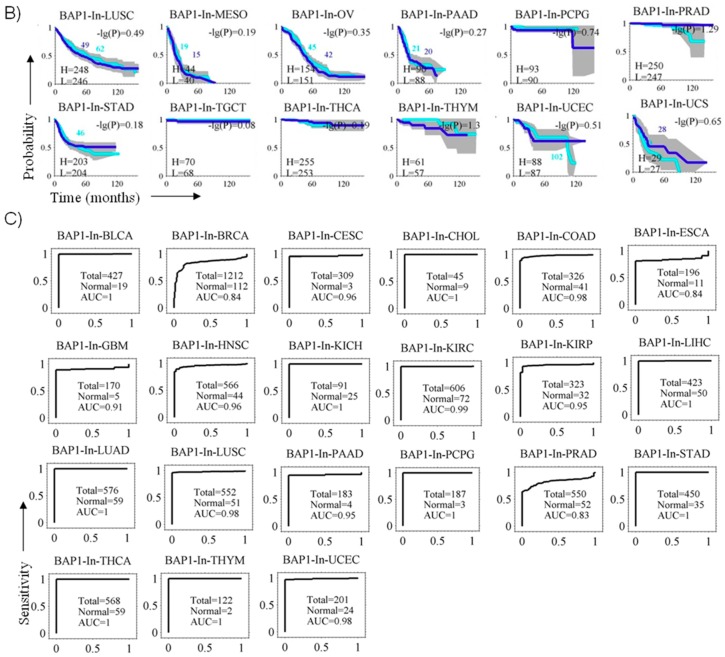
Estimation of the prognostic and diagnostic value in BAP1 gene expression in 29 cancer types (TCGA analysis). (**A**) Boxplots showing BAP1 gene expression in cancer (**C**) and in control (N) samples derived from the TCGA cohort. For each cancer type (*n* = 29), the cancer samples are divided into high- (H) and low- (L) expression types. (**B**) The survival curves of the BAP1 gene in high- (H) and low- (L) expression from different cancer types (Blue: Low expression group; Sky blue: High expression group). (**C**) ROC curves reflect the good diagnosis ability of BAP1 in various cancers.

In TCGA (The Cancer Genome Atlas), apart from UM, we identified 29 cancer types with altered BAP1 gene expression (Figure 4A). Based on high (H) or low (L) BAP1 gene expression levels, we investigated the survival difference within these two groups and found that in LGG, HNSC and PRAD, the survival difference between BAP1 high- and low- expression patients was slightly significant (–log­_10_ (*p*-value) >1.2) (Figure 4A, B). In addition, to determine the diagnostic capacity of the BAP1 gene for control and cancer samples, we used the area under the receiver operating characteristic curve (AUC). Our analysis showed that the gene expression of BAP1 is significantly different between cancer and control samples for most of the cancer types included in this study. However, the significance was moderate (AUC > 0.8) in the case of BRCA, ESCA and PRAD (Figure 4C). 

## 4. Discussion

By virtue of the complex regulation of the *BAP1* gene and its associated proteins, it can be assumed that the specific type of genetic rearrangements (mutations, polymorphisms) in this gene will lead to disease-specific functional consequences. Considering this, we conducted this study to determine the potential impact of UM-associated mutations in BAP1-related protein complexes. BAP1 is a key contributing factor to UM, and its (epi)genetic prospective has been recently discussed [33,34]. Thus, we thoroughly analyzed the genetic alterations of the *BAP1* gene and generated the mutational landscape specifically for UM. We first showed that the genomic mutations were mainly localized at the UCH domain. These mutations occur at the conserved catalytic sites, as well as at the overlapping phosphorylation residues. Interestingly, most of the mutations showed a very high evolutionary action (EA) score, which is consistent with the fact that BAP1 acts as tumor suppressor and harbors metastatic potential in combination with other factors. Our analysis also comes in agreement with previous studies which have consistently used EA scores to assess the mutational impact and to identify the driver genes in cancers [35,36,37]. In context to the functional impact of these mutations, one study has previously described the structural destabilization of BAP1 after mutations in the catalytic domain leading to aggregation, cytoplasmic sequestration and subsequent functional loss [38]. The mutations included in our study also involved the complete UCH domain. However, the consequence of these mutants at a molecular level remains unclear. Using our in silico model of the BAP1 protein, we defined the possible structural changes that could result in alterations of the protein-protein interactions. 

In UM, there has been no missense mutation described in the HBM domain. Hence, the potential consequences of the direct BAP1-HCF1 interaction pathway cannot be determined in our study. However, FOXK2 recruits BAP1 to the target genes where HCF-1 is involved in bridging the ternary complex (FOXK2-BAP1-HCF1) [39]. Our structural analysis clearly showed closer interactions of neighboring residues of the S482L mutation with the forkhead-associated domain of FOXK2. Therefore, an indirect role of HCF-1 can be speculated. 

The potential involvement of HCF-1 is also evident from the D672G mutation, which is located in the *BAP1* region essential for interactions with transcription factor YY1. Here again, YY1 requires HCF-1 to form a ternary complex with BAP1 [40]. At the C-terminus, apart from YY1 interactions, ASXL1 also interacts with the ULD domain of BAP1 to drive the H2A deubiquitination reaction [41]. In our structural analysis, the neighboring residues of mutation D672 were found to be directly involved in ASXL1-BAP1 interactions; hence, alterations in the activity of PR-DUB complex can be predicted. BAP1 harbors interacting sites for BARD1 and BRCA1 proteins. Interestingly, both of these proteins contain an N-terminal zinc RING-finger domain which confers E3 ubiquitin ligase activity to regulate the DNA damage response [42]. However, BAP1 inhibits the E3 ligase activity of BRCA1/BARD1 complex by binding to BARD1 [43]. In our study, we observed that both (BARD1 and BRCA1) interacting regions of *BAP1* were enriched with phosphorylation sites. Although the regulatory mechanisms involving phosphorylation are very widespread, it is well established that phosphorylation can modulate protein-protein interactions. The impact of enriched phosphorylation sites in our study is unclear. Since BARD1 directly interacts with BAP1, the major impact of mutations in BARD1 (E182G, G185R) as compared to BRCA1 (E602D) can be seen in the structural analysis. It is also noteworthy to mention that we found 15 phosphorylation sites solely in the UCH domain of *BAP1* and three mutations (S63C, S98R, T117R) were found to be overlapping with these sites. There is concrete evidence in the literature that disruptions of phosphorylation sites are associated with cancer [44,45]. Hence, a thorough investigation of these UM-associated mutations is warranted.

Considering that the host gene (BAP1) is mutated, it will automatically contribute to an altered expression of miRNAs located within its proximity. Therefore, we also investigated the regulatory network of miRNAs embedded within 3´ UTR of the BAP1 gene. In contrast to N-terminal mutations, C-terminal variants showed a stronger binding affinity towards these miRNAs. Besides, these miRNAs have previously been shown to be involved in multiple cancer types. Among them, the miR-125 family, which potentially acts as tumor suppressors has been reported in different types of cancer, including cutaneous melanoma [46,47]. Likewise, miR-200a-3p (renal carcinoma, pancreatic cancer), miR-423-5p (gastric cancer), miR-31-5p(Oral Cancer), miR-141-3p (rectal cancer) and miR-140-3p.1 (breast cancer) have also been implicated in different types of cancer [48,49,50,51,52,53]. Based on our study, we have observed that the regulatory network associated with BAP1-miRNAs is unique in comparison to the common miRNAs regulatory network in UM. This, in turn, suggests that the BAP1-miRNAs associated network (in combination with respective BAP1 mutations) might have an independent impact in UM. Regarding the recently identified connection between BAP1 and HDAC4 [34], it appeared beyond others also in our local gene regulatory miRNA-mediated network. 

Since the mutational load can vary depending on the tumor type, the recurrent mutation rate in genes or the gene expression itself can be a predictive marker for tumor progression. Considering this, we checked the BAP1 gene expression from the TCGA dataset and found that regardless of the mutation type, an alteration of the BAP1 gene expression can be used for the prognosis in 29 tumor types. This suggests that although BAP1 is conceptually a driver gene in UM, it might contribute globally to the cancer genome. 

## 5. Conclusions

Although BAP1 is conceptually a driver gene in UM, it might contribute through its interaction partners and its regulatory miRNA network to various aspects of cancer. In future, similar studies on this gene by focusing on other cancer types can help to define its thorough molecular consequences in cancers. From a diagnostic point of view, our work will help the clinicians to consider the role of the BAP1 gene expression in 29 cancer types, which were never discussed before.

## Supplementary Materials

The following are available online at https://www.mdpi.com/2072-6694/11/10/1600/s1, Figure S1: BAP1 variants and stability of multiprotein complexes. Simultaneous view of residue interaction network (left) and 3D structure (in silico analysis) of BAP1-associated protein complexes, such as BAP1-HCF1, BAP1-FOXK2, BAP1-ASXL1, BAP1-BRCA1-BARDI (right). UM-associated mutations are marked (red) for each protein complex, Figure S2: Phosphorylation residues in BAP1 protein are marked (red), Table S1: List of phosphorylation and kinases in BAP1 protein, Table S2: Minimum free energy (mfe) values for BAP1 and miRNAs.

## Abbreviation List

UCH	Ubiquitin carboxyl hydrolase domain
HBM	HCF1 binding motif
NLS	Nuclear localization signals
LAML	Acute Myeloid Leukemia
ACC	Adrenocortical carcinoma
BLCA	Bladder Urothelial Carcinoma
LGG	Brain Lower Grade Glioma
BRCA	Breast invasive carcinoma
CESC	Cervical squamous cell carcinoma and endocervical adenocarcinoma
CHOL	Cholangiocarcinoma
LCML	Chronic Myelogenous Leukemia
COAD	Colon adenocarcinoma
ESCA	Esophageal carcinoma
GBM	Glioblastoma multiforme
HNSC	Head and Neck squamous cell carcinoma
BAP1	BRCA1-associated protein 1
KICH	Kidney Chromophobe
KIRC	Kidney renal clear cell carcinoma
KIRP	Kidney renal papillary cell carcinoma
LIHC	Liver hepatocellular carcinoma
LUAD	Lung adenocarcinoma
LUSC	Lung squamous cell carcinoma
DLBC	Lymphoid Neoplasm Diffuse Large B-cell Lymphoma
MESO	Mesothelioma
OV	Ovarian serous cystadenocarcinoma
PAAD	Pancreatic adenocarcinoma
PCPG	Pheochromocytoma and Paraganglioma
PRAD	Prostate adenocarcinoma
STAD	Stomach adenocarcinoma
TGCT	Testicular Germ Cell Tumors
THYM	Thymoma
THCA	Thyroid carcinoma
UCS	Uterine Carcinosarcoma
UCEC	Uterine Corpus Endometrial Carcinoma
