# Supplementary material for: Mutational Landscape of the BAP1 Locus Reveals an Intrinsic Control to Regulate the miRNA Network and the Binding of Protein Complexes in Uveal Melanoma
Source: Cancers (Basel). 2019 Oct 19;11(10):1600. doi: 10.3390/cancers11101600 (PMC6826957; doi:10.3390/cancers11101600)
Supplement: Supplementary file 1 [file cancers-11-01600-s001.pdf]

Supplementary Materials: Mutational Landscape of the BAP1 Locus Reveals an Intrinsic Control to Regulate the miRNA Network and the Binding of Protein Complexes in Uveal Melanoma

Amit Sharma, Arijit Biswas, Hongde Liu, Sagnik Sen, Anoosha Paruchuri, Panagiotis Katsonis, Olivier Lichtarge, Tikam Chand Dakal, Ujjwal Maulik, M. Michael Gromiha, Sanghamitra Bandyopadhyay, Michael Ludwig, Frank G. Holz, Karin U. Loeffler and Martina C. Herwig–Carl

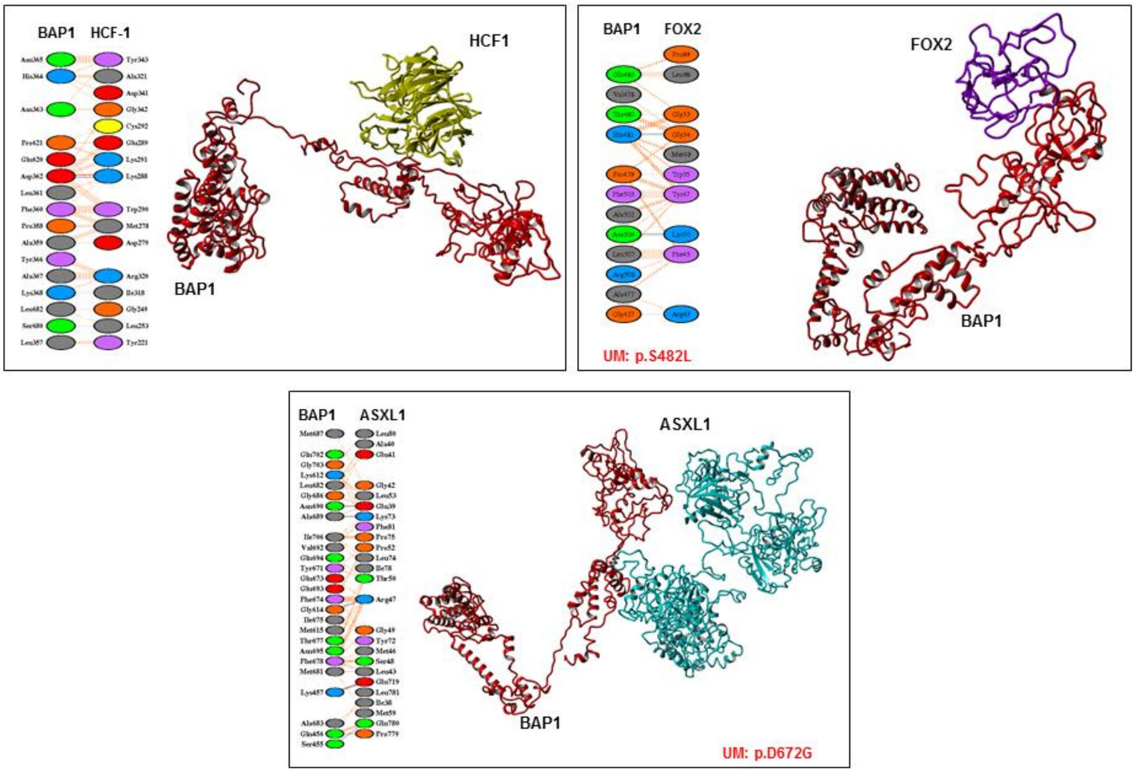

Figure S1. BAP1 variants and stability of multiprotein complexes**.** Simultaneous view of residue interaction network (left) and 3D structure (*in silico analysis*) of BAP1−associated protein complexes such as BAP1−HCF1, BAP1−FOXK2 and BAP1−ASXL1 (right) is shown. UM associated mutations are marked (red) for each protein complex.

BAP1 Protein Sequence

```
MNKGWLELESDPGLFTLLVEDFGVKGVQVEEIYDLQSKCQGPVYGFIFLF     050
KWIEERRSRRKVSTLVDDTSVIDDDIVNNMFFAHQLIPNSCATHALLSVL     100
LNCSSVDLGPTLSRMKDFTKGFSPESKGYAIGNAPELAKAHNSHARPEPR     150
HLPEKQNGLSAVRTMEAFHFVSYVPITGRLFELDGLKVYPIDHGPWGEDE     200
EWTDKARRVIMERIGLATAGEPYHDIRFNLMAVVPDRRIKYEARLHVLKV     250
NRQTVLEALQQLIRVTQPELIQTHKSQESQLPEESKSASNKSPLVLEANR     300
APAASEGNHTDGAEEAAGSCAQAPSHSPPNKPKLVVKPPGSSLNGVHPNP     350
TPIVQRLPAFLDNHNYAKSPMQEEEDLAAGVGRSRVPVRPPQQYSDDEDD     400
YEDDEEDDVQNTNSALRYKGKGTGKPGALSGSADGQLSVLQPNTINVLAE     450
KLKESQKDLSIPLSIKTSSGAGSPAVAVPTHSQPSPTPSNESTDTASEIG     500
SAFNSPLRSPIRSANPTRPSSPVTSHISKVLFGEDDSLLRVDCIRYNRAV     550
RDLGPVISTGLLHLAEDGVLSPLALTEGGKGSSPSIRPIQGSQGSSSPVE     600
KEVVEATDSREKTGMVRPGEPLSGEKYSPKELLALLKCVEAEIANYEACL     650
KEEVEKRKKFKIDDQRRTHNYDEFICTFISMLAQEGMLANLVEQNISVRR     700
RQGVSIGRLHKQRKPDRRKRSRPYKAKRQ                          750
```

Figure S2. Phosphorylation residues in BAP1 protein are marked (red).

Table S1. List of phosphorylation and kinases in BAP1 protein.

# Sequence	#	x	Context	Score	Kinase	Answer
# ---------------	-----------	---	------------	--------	-------------	-----
# Sequence	33	Y	VEEIYDLQS	0.645	unsp	YES
# Sequence	37	S	YDLQSKCQG	0.673	unsp	YES
# Sequence	37	S	YDLQSKCQG	0.662	PKC	YES
# Sequence	58	S	EERRSRRKV	0.996	unsp	YES
# Sequence	58	S	EERRSRRKV	0.569	PKC	YES
# Sequence	63	S	RRKVSTLVD	0.982	unsp	YES
# Sequence	63	S	RRKVSTLVD	0.834	PKA	YES
# Sequence	63	S	RRKVSTLVD	0.571	PKG	YES
# Sequence	63	S	RRKVSTLVD	0.53	cdc2	YES
# Sequence	63	S	RRKVSTLVD	0.521	RSK	YES
# Sequence	64	T	RKVSTLVDD	0.841	unsp	YES
# Sequence	64	T	RKVSTLVDD	0.518	PKC	YES
# Sequence	69	T	LVDDTSVID	0.532	CKII	YES
# Sequence	70	S	VDDTSVIDD	0.995	unsp	YES
# Sequence	70	S	VDDTSVIDD	0.554	CKI	YES
# Sequence	70	S	VDDTSVIDD	0.526	CKII	YES
# Sequence	98	S	HALLSVLLN	0.854	PKC	YES
# Sequence	105	S	LNCSSVDLG	0.538	cdc2	YES
# Sequence	119	T	MKDFTKGFS	0.743	PKC	YES
# Sequence	119	T	MKDFTKGFS	0.501	CKI	YES
# Sequence	123	S	TKGFSPESK	0.993	unsp	YES
# Sequence	123	S	TKGFSPESK	0.521	p38MAPK	YES
# Sequence	123	S	TKGFSPESK	0.505	GSK3	YES
# Sequence	126	S	FSPESKGYA	0.557	CKI	YES
# Sequence	126	S	FSPESKGYA	0.535	unsp	YES
# Sequence	129	Y	ESKGYAIGN	0.857	unsp	YES
# Sequence	129	Y	ESKGYAIGN	0.52	INSR	YES
# Sequence	143	S	KAHNSHARP	0.559	unsp	YES
# Sequence	160	S	QNGLSAVRT	0.814	unsp	YES
# Sequence	160	S	QNGLSAVRT	0.526	cdc2	YES
# Sequence	164	T	SAVRTMEAF	0.508	PKC	YES
# Sequence	177	T	YVPITGRLF	0.592	unsp	YES
# Sequence	177	T	YVPITGRLF	0.521	PKC	YES
# Sequence	203	T	DEEWTDKAR	0.699	unsp	YES
# Sequence	223	Y	AGEPYHDIR	0.937	unsp	YES
# Sequence	254	T	VNRQTVLEA	0.941	unsp	YES
# Sequence	254	T	VNRQTVLEA	0.617	PKA	YES
# Sequence	266	T	LIRVTQPEL	0.562	DNAPK	YES
# Sequence	266	T	LIRVTQPEL	0.562	PKA	YES
# Sequence	266	T	LIRVTQPEL	0.505	PKG	YES
# Sequence	273	T	ELIQTHKSQ	0.672	unsp	YES
# Sequence	273	T	ELIQTHKSQ	0.619	PKC	YES
# Sequence	276	S	QTHKSQESQ	0.991	unsp	YES
# Sequence	276	S	QTHKSQESQ	0.619	ATM	YES
# Sequence	276	S	QTHKSQESQ	0.516	DNAPK	YES

# Sequence	279	S	KSQESQLPE	0.545	DNAPK	YES
# Sequence	279	S	KSQESQLPE	0.531	unsp	YES
# Sequence	279	S	KSQESQLPE	0.522	CKII	YES
# Sequence	287	S	EESKSASNK	0.939	unsp	YES
# Sequence	287	S	EESKSASNK	0.552	cdc2	YES
# Sequence	289	S	SKSASNKSP	0.987	unsp	YES
# Sequence	289	S	SKSASNKSP	0.783	PKC	YES
# Sequence	289	S	SKSASNKSP	0.524	cdc2	YES
# Sequence	292	S	ASNKSPLVL	0.908	unsp	YES
# Sequence	292	S	ASNKSPLVL	0.519	p38MAPK	YES
# Sequence	305	S	APAASEGNH	0.969	unsp	YES
# Sequence	310	T	EGNHTDGAE	0.645	CKII	YES
# Sequence	319	S	EAAGSCAQA	0.563	PKC	YES
# Sequence	327	S	APSHSPPNK	0.867	unsp	YES
# Sequence	327	S	APSHSPPNK	0.521	GSK3	YES
# Sequence	342	S	PPGSSLNGV	0.503	PKA	YES
# Sequence	351	T	HPNPTPIVQ	0.639	cdk5	YES
# Sequence	366	Y	DNHNYAKSP	0.871	unsp	YES
# Sequence	369	S	NYAKSPMQE	0.617	unsp	YES
# Sequence	369	S	NYAKSPMQE	0.539	CKII	YES
# Sequence	395	S	PQQYSDDED	0.954	unsp	YES
# Sequence	395	S	PQQYSDDED	0.716	CKII	YES
# Sequence	401	Y	DEDDYEDDE	0.993	unsp	YES
# Sequence	401	Y	DEDDYEDDE	0.569	SRC	YES
# Sequence	401	Y	DEDDYEDDE	0.536	INSR	YES
# Sequence	418	Y	SALRYKGKG	0.599	unsp	YES
# Sequence	423	T	KGKGTGKPG	0.761	unsp	YES
# Sequence	423	T	KGKGTGKPG	0.551	PKC	YES
# Sequence	423	T	KGKGTGKPG	0.528	PKG	YES
# Sequence	430	S	PGALSGSAD	0.835	unsp	YES
# Sequence	430	S	PGALSGSAD	0.538	PKC	YES
# Sequence	432	S	ALSGSADGQ	0.541	cdc2	YES
# Sequence	455	S	KLKESQKDL	0.998	unsp	YES
# Sequence	455	S	KLKESQKDL	0.625	ATM	YES
# Sequence	460	S	QKDLSIPLS	0.553	unsp	YES
# Sequence	464	S	SIPLSIKTS	0.889	unsp	YES
# Sequence	464	S	SIPLSIKTS	0.761	PKC	YES
# Sequence	469	S	IKTSSGAGS	0.807	unsp	YES
# Sequence	469	S	IKTSSGAGS	0.716	PKC	YES
# Sequence	473	S	SGAGSPAVA	0.909	unsp	YES
# Sequence	473	S	SGAGSPAVA	0.567	cdk5	YES
# Sequence	473	S	SGAGSPAVA	0.519	GSK3	YES
# Sequence	480	T	VAVPTHSQP	0.651	PKC	YES
# Sequence	482	S	VPTHSQPSP	0.645	DNAPK	YES
# Sequence	482	S	VPTHSQPSP	0.553	ATM	YES
# Sequence	485	S	HSQPSPTPS	0.985	unsp	YES
# Sequence	485	S	HSQPSPTPS	0.546	p38MAPK	YES
# Sequence	485	S	HSQPSPTPS	0.538	cdk5	YES
# Sequence	485	S	HSQPSPTPS	0.503	GSK3	YES
# Sequence	487	T	QPSPTPSNE	0.526	cdk5	YES
# Sequence	489	S	SPTPSNEST	0.987	unsp	YES
# Sequence	492	S	PSNESTDTA	0.863	unsp	YES
# Sequence	492	S	PSNESTDTA	0.539	CKII	YES
# Sequence	493	T	SNESTDTAS	0.547	CKII	YES
# Sequence	493	T	SNESTDTAS	0.514	CKI	YES
# Sequence	495	T	ESTDTASEI	0.567	unsp	YES
# Sequence	497	S	TDTASEIGS	0.878	unsp	YES
# Sequence	497	S	TDTASEIGS	0.528	cdc2	YES
# Sequence	505	S	SAFNSPLRS	0.833	unsp	YES
# Sequence	505	S	SAFNSPLRS	0.7	cdk5	YES
# Sequence	505	S	SAFNSPLRS	0.517	GSK3	YES
# Sequence	509	S	SPLRSPIRS	0.972	unsp	YES
# Sequence	509	S	SPLRSPIRS	0.707	cdk5	YES
# Sequence	509	S	SPLRSPIRS	0.531	p38MAPK	YES
# Sequence	509	S	SPLRSPIRS	0.528	GSK3	YES
# Sequence	513	S	SPIRSANPT	0.722	unsp	YES
# Sequence	513	S	SPIRSANPT	0.592	PKG	YES
# Sequence	517	T	SANPTRPSS	0.743	PKC	YES
# Sequence	517	T	SANPTRPSS	0.504	GSK3	YES
# Sequence	520	S	PTRPSSPVT	0.678	unsp	YES

# Sequence	520	S	PTRPSSPVT	0.585	PKC	YES
# Sequence	520	S	PTRPSSPVT	0.533	cdc2	YES
# Sequence	521	S	TRPSSPVTS	0.994	unsp	YES
# Sequence	521	S	TRPSSPVTS	0.656	cdk5	YES
# Sequence	521	S	TRPSSPVTS	0.538	GSK3	YES
# Sequence	521	S	TRPSSPVTS	0.507	p38MAPK	YES
# Sequence	524	T	SSPVTSHIS	0.529	PKC	YES
# Sequence	525	S	SPVTSHISK	0.866	unsp	YES
# Sequence	537	S	GEDDSLLRV	0.511	cdc2	YES
# Sequence	558	S	GPVISTGLL	0.524	PKA	YES
# Sequence	559	T	PVISTGLLH	0.529	cdc2	YES
# Sequence	571	S	DGVLSPLAL	0.584	p38MAPK	YES
# Sequence	583	S	GKGSSPSIR	0.713	unsp	YES
# Sequence	583	S	GKGSSPSIR	0.562	cdk5	YES
# Sequence	585	S	GSSPSIRPI	0.819	unsp	YES
# Sequence	585	S	GSSPSIRPI	0.664	PKC	YES
# Sequence	592	S	PIQGSQGSS	0.663	unsp	YES
# Sequence	592	S	PIQGSQGSS	0.63	DNAPK	YES
# Sequence	592	S	PIQGSQGSS	0.623	PKC	YES
# Sequence	592	S	PIQGSQGSS	0.612	ATM	YES
# Sequence	596	S	SQGSSSPVE	0.613	PKC	YES
# Sequence	596	S	SQGSSSPVE	0.535	CKII	YES
# Sequence	597	S	QGSSSPVEK	0.997	unsp	YES
# Sequence	597	S	QGSSSPVEK	0.506	cdc2	YES
# Sequence	607	T	VVEATDSRE	0.541	CKII	YES
# Sequence	609	S	EATDSREKT	0.956	unsp	YES
# Sequence	613	T	SREKTGMVR	0.896	unsp	YES
# Sequence	623	S	GEPLSGEKY	0.956	unsp	YES
# Sequence	623	S	GEPLSGEKY	0.533	ATM	YES
# Sequence	627	Y	SGEKYSPKE	0.886	unsp	YES
# Sequence	628	S	GEKYSPKEL	0.997	unsp	YES
# Sequence	677	T	EFICTFISM	0.519	PKC	YES
# Sequence	680	S	CTFISMLAQ	0.504	cdc2	YES
# Sequence	697	S	EQNISVRRR	0.804	PKC	YES
# Sequence	705	S	RQGVSIGRL	0.869	unsp	YES
# Sequence	721	S	RRKRSRPYK	0.877	unsp	YES
# Sequence	721	S	RRKRSRPYK	0.693	PKA	YES
# Sequence	721	S	RRKRSRPYK	0.682	PKG	YES
# Sequence	721	S	RRKRSRPYK	0.561	RSK	YES

Table S2. Binding minimum free energy of BAP1 variants with miRNAs.

Heading title	mi-RNA-31	mi-RNA-125a	mi-RNA-125b	mi-RNA-200b	mi-RNA-423	mi-RNA-505	mi-RNA-140
**BAP1-WT**	−68.4	−88.7	−83.2	−100.4	−82.9	−83.2	−91.8
**M1T**	−68.4	−88.7	−83.2	−100.4	−82.9	−83.2	−91.8
**M1I**	−68.4	−88.7	−83.2	−100.4	−82.9	−83.2	−91.8
**G45R**	−59.5	−81.3	−74.7	−85.1	−79.5	−74.4	−82.0
**S63C**	−68.4	−88.7	−83.2	−100.4	−82.9	−83.2	−91.8
**Q85P**	−62.0	−88.7	−83.2	−100.4	−82.9	−83.2	−91.8
**C91G**	−68.4	−88.7	−83.2	−100.4	−82.9	−83.2	−91.8
**L97Q**	−68.4	−88.7	−83.2	−100.4	−82.9	−83.2	−91.8
**S98R**	−65.2	−83.7	−76.3	−91.8	−82.9	−83.2	−91.8
**L100P**	−68.4	−88.7	−83.2	−100.4	−82.9	−83.2	−91.8
**L112R**	−68.4	−88.7	−83.2	−100.4	−82.9	−83.2	−91.8
**M115V**	−68.4	−88.7	−83.2	−100.4	−82.9	−83.2	−91.8
**G128R**	−64.8	−80.7	−83.2	−100.4	−79.5	−75.5	−87.8
**H141R**	−64.5	−81.3	−75.0	−91.0	−79.5	−83.2	−86.0
**R146K**	−68.4	−88.7	−83.2	−100.4	−82.9	−83.2	−91.8
**H169P**	−68.4	−88.7	−83.2	−100.4	−82.9	−83.2	−91.8
**S172R**	−64.4	−87.7	−80.2	−90.4	−79.9	−82.2	−90.4
**Y173C**	−68.4	−88.7	−83.2	−100.4	−82.9	−83.2	−91.8
**P175R**	−68.4	−88.7	−83.2	−100.4	−82.9	−83.2	−91.8
**T177R**	−68.4	−88.7	−83.2	−100.4	−82.9	−83.2	−91.8
**L180P**	−68.4	−88.7	−83.2	−100.4	−82.9	−83.2	−91.8
**E182D**	−68.4	−88.7	−83.2	−100.4	−82.9	−83.2	−91.8

**E182G**	−68.4	−88.7	−83.2	−100.4	−82.9	−83.2	−91.8
**G185R**	−62.4	−82.5	−81.1	−97.4	−78.5	−80.2	−89.7
**E212D**	−68.4	−88.7	−83.2	−100.4	−82.9	−83.2	−91.8
**N229D**	−68.4	−88.7	−83.2	−100.4	−82.9	−83.2	−91.8
**S278L**	−68.4	−88.7	−83.2	−100.4	−82.9	−83.2	−91.8
**S280T**	−68.4	−88.7	−83.2	−100.4	−82.9	−83.2	−91.8
**D400Y**	−68.4	−88.7	−83.2	−100.4	−82.9	−83.2	−91.8
**N443T**	−65.2	−88.7	−75.6	−100.4	−82.9	−83.2	−91.8
**S482L**	−68.4	−88.7	−83.2	−100.4	−82.9	−83.2	−91.8
**L570V**	−68.4	−88.7	−83.2	−100.4	−82.9	−83.2	−91.8
**E577N**	−68.4	−88.7	−83.2	−100.4	−82.9	−83.2	−91.8
**E577Q**	−68.4	−88.7	−83.2	−100.4	−82.9	−83.2	−91.8
**E602D**	−27.4	−81.3	−75.6	−91.8	−82.9	−75.5	−87.8
**D672G**	−26.3	−80.6	−75.6	−89.9	−79.4	−74.7	−83.1

© 2019 by the authors. Licensee MDPI, Basel, Switzerland. This article is an open access article distributed under the terms and conditions of the Creative Commons Attribution (CC BY) license (http://creativecommons.org/licenses/by/4.0/).
